# Economic interventions to improve population health: a scoping study of systematic reviews

**DOI:** 10.1186/s12889-016-3119-5

**Published:** 2016-07-07

**Authors:** Mishal S. Khan, Bernie Y. Guan, Jananie Audimulam, Francisco Cervero Liceras, Richard J. Coker, Joanne Yoong

**Affiliations:** Saw Swee Hock School of Public Health, National University of Singapore, Tahir Foundation Building, National University of Singapore, 12 Science Drive 2, #10-01, Singapore, 117549 Singapore; Communicable Diseases Policy Research Group, London School of Hygiene and Tropical Medicine, Keppel St, London, WC1E 7HT UK; Faculty of Public Health, Mahidol University, 420/1 Ratchawithi RD, Ratchathewi District Bangkok, 10400 Thailand; Center for Economic and Social Research, University of Southern California, 635 Downey Way, VPD, Los Angeles, CA 90089 USA

**Keywords:** Economic interventions, Public health, Behavior change, Review

## Abstract

**Background:**

Recognizing the close relationship between poverty and health, national program managers, policy-makers and donors are increasingly including economic interventions as part of their core strategies to improve population health. However, there is often confusion among stakeholders about the definitions and operational differences between distinct types of economic interventions and financial instruments, which can lead to important differences in interpretation and expectations.

**Methods:**

We conducted a scoping study to define and clarify concepts underlying key economic interventions - price interventions (taxes and subsidies), income transfer programs, incentive programs, livelihood support programs and health-related financial services – and map the evidence currently available from systematic reviews.

**Results:**

We identified 195 systematic reviews on economic interventions published between 2005 and July 2015. Overall, there was an increase in the number of reviews published after 2010. The majority of reviews focused on price interventions, income transfer programs and incentive programs, with much less evidence available from systematic reviews on livelihood support programs and health-related financial services. We also identified a lack of evidence on: health outcomes in low income countries; unintended or perverse outcomes; implementation challenges; scalability and cost-effectiveness of economic interventions.

**Conclusions:**

We conclude that while more research is clearly needed to assess suitability and effectiveness of economic interventions in different contexts, before interventions are tested and further systematic reviews conducted, a consistent and accurate understanding of the fundamental differences in terminology and approaches is essential among researchers, public health policy makers and program planners.

## Background

It is widely acknowledged that conditions of poverty and ill-health exacerbate each other [1]. Poor health increases expenditure on medical care and reduces productivity and hence income [2, 3]. The constraints of low income in turn affect health negatively, through financial barriers to accessing good quality medical care, dietary deprivation and exposure to environmental risk factors such as poor sanitation and over-crowding.

Recognizing the close relationship between poverty and ill-health, national program managers, policy-makers and donors are increasingly including economic interventions as part of their core strategies to improve population health. For example, 19 (73 %) out of 26 tuberculosis (TB) control proposals approved by the Global Fund in 2010 included either direct or indirect economic support [4]. Health policy and research on issues as diverse as smoking during pregnancy and better use of health services increasingly indicates interest in introducing economic interventions [5, 6]. However, there is often confusion among public health practitioners about the definitions and operational differences between distinct types of interventions and financial instruments, which are often discussed interchangeably as if they all have the same objectives and approach [7].

Such ambiguity can lead to important differences in interpretation and expectations. With the breadth and complexity of these applications in mind, we therefore conducted a scoping review to identify different types of economic interventions that public health policy makers and program planners are most likely to encounter or consider implementing, to articulate differences in concepts underlying these different interventions and their applications, and to map the relevant evidence currently available. The purpose of this review is to clarify these terms for policymakers and practitioners and help to assess the value and feasibility of more targeted future research, including future systematic reviews.

## Methods

Based on our study objectives, we adopted best-practice principles of rapid (scoping) review designs based on the methodology outlined by various authors [8–11]. To identify relevant studies in a comprehensive but feasible manner, we used an iterative approach.

Following a series of exploratory searches in PubMed and Google Scholar, we determined that the specific terms “economic” and “intervention” would yield results too broad and diverse to be useful, including a large number of irrelevant results related to economic evaluation but not economic interventions, defined as interventions that fundamentally target the economic status or decision making of households with respect to health and healthcare. Based on the abstracts and study descriptions from this initial exploration, we extracted an initial set of the most common terms used to describe economic interventions. These included: tax, subsidy, cash transfer, income transfer, bonus, pay-for-performance, micro-credit and micro-finance.

Based on the underlying mechanisms of these economic interventions, these terms were then grouped into five main categories: price-based interventions (taxes and subsidies), income transfer programs, incentive programs, livelihood support programs and health-related financial services. We then further expanded the list of search terms under each category, paying particular attention to including alternative phrases that refer to the same economic intervention, such as income support/income supplement/financial supplement.

We conducted five searches of systematic reviews published between 1st January 2015 and 10th July 2015 using PubMed (Medline) and The Cochrane Library (2015 July). We searched for papers containing ‘health’ in any field plus any of the final search terms detailed in Table 1 in the title or abstract, with the following filters: English language and Humans.

**Table 1:** Concepts and search terms combined with ‘health’

Economic interventions	Definition	Search terms
Price Interventions - Taxes and Subsidies	Interventions that target prices for goods and services paid/received by households or firms	“tax”, “subsidy”, “subsidized”, “subsidies”, “penalty”, “penalties”, “voucher”, “vouchers”
Income Transfer Programs	Interventions that transfer resources directly to households	“income support”, “financial supplement”, “income supplement”, “fina ncial assistance”, “welfare benefits”, “social security”, “cash assistance”, “income transfer”, “asset transfer”, “pensions”, “welfare payments”, “cash transfer”, “cash support”, “bonus”, “economic support”
Incentive Programs	Interventions that provide rewards or penalties to motivate specific behaviours/outcomes	“results-based financing”, “pay for performance”, “performance pay”, “performance incentives”, “financial incentives”, “activity based funding”, “provider payment”
Livelihood Support Programs	Interventions that provide resources or skills to support income generation	“livelihood support”, “entrepreneurship training”, “business training”, “vocational skills training”, “vocational training”
Health-related Financial Services	Interventions that provide mechanisms for managing resource allocations and financial risks.	“microcredit”, “microlending”, “microloans”, “microfinance”, “loans”, “lending”, “microinsurance”

**Fig. 1:**
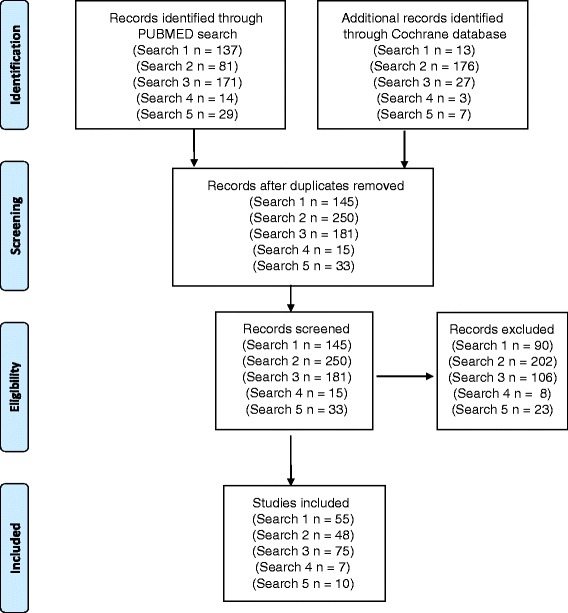
Process and results of literature search

Three researchers (BG, JA, MK) conducted the literature review (Fig. 1). Results from the bibliographic databases were merged and duplicates were removed. The literature review was conducted in sequential stages. First, two reviewers (BG and JA) independently screened the search results by title and abstract to identify relevant papers for inclusion. If the decision for inclusion could not be made on the basis of information presented in the title and abstract, the full text was reviewed. The following inclusion criteria were applied: systematic review; involved assessment of one of more economic interventions listed above; impact on any aspect of population health studied. Exclusion criteria were: protocol only; assessed impact of incentives on participation in trials with no specific health outcome. Once both reviewers had determined eligibility independently, their assessments on each paper were compared, and any disagreements were resolved by consulting the third researcher (MK).

For each category of intervention, given the heterogeneity of interventions, outcome measurements, and settings, it was not appropriate to analyze the quality of reviews or evidence on impact. Instead, we used the reviews to address our first research objective by refining a definition of economic interventions and clarifying the underlying concepts, and highlighting relevant examples. We then addressed our second research objective by undertaking a mapping exercise where we focused on the size and chronology of the literature. We analyzed changes in the number of systematic reviews for each economic intervention over our ten year search period, and highlighted the main health outcomes that systematic reviews had focused on, thereby indicating areas where evidence of impact available for policy makers and programme planners is available.

## Results

Based on our review, we further refined the following working definition of “economic intervention for health”: an intervention primarily designed to improve health outcomes by addressing the underlying determinants of demand or supply for health and health care – tastes/preferences (such as advertising), prices (such as taxes or subsidies), income (such as welfare programs or programs that enhance the ability to earn income), credit (such as small loans) and uncertainty (such as insurance). Interventions that correct imperfections or asymmetries in relevant information (such as provider report cards) may also fall under this (wide) technical definition.

We found that economic interventions may be the central part of a health program (such as health insurance for the poor), or undertaken as ancillary to a treatment program (such as part of an overall socioeconomic support package for patients). Macro-level economic interventions take place at the level of markets for specific goods and services such as tobacco taxation policies, national insurance programs or fiscal policies affecting public health spending. Meso-level economic interventions operate at the level of institutions, such as grants or financing facilities provided to national disease programs for certain drugs or vaccines, while micro-level economic interventions target individual households, patients and providers. We first provide a refined definition of economic interventions in a health-related context, and then summarize findings from our mapping of evidence from systematic reviews.

### Price interventions - taxes and subsidies

Governments and donors can intervene directly in markets for healthcare goods and services to exercise control over prices. For instance, in many critical services such as childhood immunization and national disease programs such as HIV or TB are typically provided by the public sector at reduced or no charge. Subsidy programs may be universal or further targeted to specific groups (also known as dual-pricing) via ration cards, stamps or vouchers for goods and services [12–14]. For instance, voucher schemes have been introduced in a number of developing countries to encourage low-income women to take up maternity services, including antenatal care and delivery [15].

Taxes and subsidies may also be deployed with the objective of health promotion outside the healthcare sector. Taxes on tobacco are widely regarded as a leading instrument for tobacco control worldwide [16]. On the other hand, subsidies for nutrition, education and public housing are often introduced for public health purposes. One of the largest examples of a national subsidy program is the Public Distribution System, which has provided price-controlled food and household items to low-income households in India for decades with the objective of preventing large-scale food insecurity and malnutrition [17].

### Income transfer programs

Transfers refer to the redistribution of resources from one party to another. Income transfer programs aim to improve health by providing resources directly to targeted individuals or households. Income transfer programs may also be referred to as grants or economic support.

Cash transfer programs include income support schemes that enable poor or otherwise vulnerable households to spend more on nutrition, healthcare, living conditions and education, all of which lead to better health in the short and long term. For example, South Africa has two well-studied programs, the Old Age Pension and the Child Support Grant (CSG), that provide monthly cash transfers to older adults and low-income primary caregivers of children respectively. Evaluation studies have found that receiving both types of support are associated with significant nutritional improvements in children [18, 19]. Apart from these more traditional social protection programs, cash transfer programs may also be intended to mitigate specific health-related loss of income or increased out-of-pocket expense due to a health condition or its treatment, such as disability benefits or travel stipends for patients [20].

Income transfer programs may also provide in-kind benefits rather than cash. The key distinction is that in-kind transfers restrict consumption to specific health-related goods and services [21, 22]. Examples of large-scale welfare programs with in-kind benefits include the provision of free school meals in India [23], and the universal program of nutritional supplementation for all adults over 70 in Chile [24]. Other common in-kind transfer programs include the provision of free food baskets to HIV or TB patients and their families. Food baskets are received on the condition that the patient visits a health centre to collect drugs for the next month of treatment. In theory such schemes can improve treatment outcomes not only by encouraging adherence to medication, but also through only improvements in diet which can enhance the impact of medication [25].

Subsidies and income transfers focus on relative prices and resource constraints respectively, but in some cases the distinction may not always be clear to practitioners. In-kind transfers may be thought of as effectively fully subsidizing the goods and services provided and vice versa – for example, food stamp programs are often referred to as both subsidies as well as in-kind transfer programs. In practice, programs that emphasize the direct delivery of benefits to needy recipients tend to be referred to as transfer programs, while programs that focus on transactions for specific goods and services tend to be referred to as subsidy programs.

### Incentive programs

Provider and patient incentive programs are designed to reward or penalize specific and measurable actions or outcomes, independent of recipient needs [26]. On the provider side, performance incentives link rewards to patient outcomes at the individual or institutional level. One of the largest such experiments involving pay-for-performance or P4P is the United Kingdom’s Quality and Outcomes Framework [27]. Introduced in 2004, this scheme links a significant fraction of individual physicians’ income to performance on a number of clinical as well as organizational measures. In 2005, Rwanda attempted a similar experience with pay-for-performance based on a series of output indicators related to the quantity and quality of maternal and child care [28, 29]. For patients, incentives typically involve rewards for compliance with specific health behaviors or health goals such as medication adherence or preventive healthcare [30, 31]. For instance, Volpp et al. have described an intervention providing smokers with money for completion of a cessation course as well as performance on subsequent biomarker-based tests of smoking cessation [32].

An important type of intervention that combines financial incentives with an income transfer program is the *conditional cash transfer (CCT).* Use of CCTs - in which targeted recipients are given transfers only if they undertake specified behaviors or meet specific goals related to health [33], including adherence to medical treatments, completing vaccination schedules, participating in health education programs, and utilizing essential health services- are becoming increasingly popular. What sets CCT programs apart from other financial incentive programs is not their basic concept but the history of their evolution - building on traditional social protection programs, with a strong incentive to ensure compliance with health-related behaviors embedded in their design. The flagship example is the Mexican program *Progresa*, currently known as *Oportunidades* [34, 35], which links bimonthly transfers to low-income households to children’s school attendance and visits to health care facilities. These have become more widespread in the past decade in middle-income countries in Latin America, Africa and Asia Pacific [36].

The design of incentive programs can take several forms, including lotteries, prizes and tournaments [37–39]. Incentives may be positive or negative – disincentives such as fines may be levied for poor performance or unhealthy behavior. Although punitive programs are less typical for practical and ethical reasons, an innovative and increasingly common design uses *commitment devices* where individuals voluntarily commit to incurring penalties for failing to meet self-imposed goals [40, 41]. Commitment devices have been used effectively in the context of weight-loss and smoking cessation [42]. These incentives can be monetary (such as pay-for-performance or fines as described above), in-kind (earning points for redeemable gifts in wellness programs or foregoing a particularly enjoyable activity) or even intangible (such as the gain or loss of professional or social recognition) [43]. A comprehensive approach might even consider patient-targeted incentives in conjunction with provider-targeted incentives although we also note that incentive programs may not be limited to patients or providers alone [32]. For instance, in China, Miller et al. showed that financial incentive programs for school principals were effective at reducing anemia prevalence among schoolchildren [44].

### Livelihood support programs

Livelihood support programs aim to also improve health by increasing household income, albeit by enhancing their ability to earn it for themselves. Such interventions may include skill-building, employment matching or general entrepreneurship training, or transfers of income-generating assets or investment goods. This is in contrast to income transfer programs, which provide their beneficiaries with unearned resources for consumption spending.

Livelihood support can also be provided in the form of microcredit, or credit services that are made available to individuals who may not qualify for traditional retail or commercial bank loans. Microcredit loans can be designed with various features that enable financial institutions to expand access to underserved groups, including use of group-based liability to offset collateral requirements, small loan sizes, frequent repayment schedules and borrower training/counseling.

Like income transfers, microcredit programs for livelihood support may be rolled out with an anti-poverty mandate that implicitly includes health improvements, or specifically be provided to patient populations that have difficulty accessing credit due to stigma, or have increased need for enterprise support [45]. Examples of the latter include microcredit facilities for the poorest TB-affected households in Lima, Peru [46] or women living with HIV/AIDS in Haiti [45]. The range of household livelihood support programs in current practice is illustrated by the example of integrated HIV care and livelihood programs (IHLP), which stem from the need to provide long-term sustainable support to patients rather than term-limited cash benefits [47]. In Uganda, IHLPs typically cover small-enterprise development assistance, or agricultural support, and include training, consultation as well as grants of cash, seeds, livestock or equipment and microcredit programs for HIV patients [47].

Finally, we note that these interventions apply to providers as well: livelihood support for healthcare entrepreneurs may also be seen as a contribution to population health [48]. For instance, providing training or support to small pharmacies or clinics may allow expansion of businesses, and hence increase access to healthcare services.

### Health-related financial services

The final category of interest relates to programs that provide financial services related to healthcare, including savings, loans and insurance. While income or livelihood support programs provide households with resources or the means to produce them, financial services allow households to manage resource allocations and risks.

Medical savings accounts or health savings accounts are savings accounts dedicated to health expenditure. Examples of this include Singapore’s national Medisave program, as well as new experiments with medical savings account provision in China. Savings products may be combined with incentives or penalties connected to health as well. For instance, the CARES program for smoking cessation in the Philippines combined a savings account with a commitment device to incentivize ex-smokers to abstain [42].

Loan products aim to alleviate credit or liquidity constraints rather than budget constraints. These may range from hire-purchase schemes for expensive equipment, to emergency community or hospital-based revolving credit lines that can be used to support unexpected medical expenditures to microcredit schemes set up for the purchase of specific health-related items. As microcredit providers increasingly leverage their platforms to provide healthcare and social services, and vice versa, such schemes may become more prevalent. For instance, Tarozzi et al. describe the offer of microcredit by BISWA, a provider in India, to support the purchase and maintenance of bed-nets in malaria-endemic Orissa [49]. Loans allow expansion of access to borrowers who simply do not have sufficient cash on hand but are fundamentally able to repay their debts, while maintaining cost-recovery from a program perspective.

Insurance products provide benefits in the event of illness, in exchange for regular premium payments, allowing households to better manage the uncertainty associated with health, by effectively pooling their unpredictable individual risks with that of other households in the scheme. Health insurance helps remove financial barriers to care in unforeseen emergencies and mitigating the impact of catastrophic health expenditures. With the expansion of universal coverage, health insurance programs are increasingly being rolled out among populations in low and middle-income countries.

However, additional financial protection may still be required to support groups that tend to be underserved by mainstream commercial or social insurance schemes [50]. These may include communities where high operating costs, poor information and other barriers prevent insurance markets from functioning, or informal sector workers in countries where most health insurance is employer based. Such programs may include specially subsidized access to a national health insurance scheme or community-managed schemes, organized either through local government or non-governmental bodies, with a cooperative, mutual or self-help element [51]. A subcategory of such programs, *microinsurance* generally refers to low-premium, low-benefit schemes for the poor, involving community-based risk-pooling and management [52]. One example is the Yeshashvini CBHI project in Karnataka, India, aimed at cooperative farmers and informal sector workers [53].

### Differences in evidence from systematic reviews

Our literature review is summarized in Fig. 1. Our five searches retrieved 658 papers. After removing duplicates we were left with 624 unique manuscripts of which 195 met the inclusion criteria (Fig. 1). Overall, our analysis shows an increase in the number of systematic reviews after 2010. Of 195 eligible systematic reviews, the vast majority (178, 91 %) assessed the following three economic interventions: taxes and subsidies; income transfer programs and incentive programs.

Under price interventions (taxes and subsidies), we identified 55 relevant reviews out of 145 retrieved from the search; the majority of which have been published after 2009. The predominant health areas that have been covered by systematic reviews on this topic include: addressing substance abuse and alcohol and tobacco control; changes in nutritional behavior and maternal and child health. We identified 48 reviews on income transfer programs; keys areas of focus include: rehabilitation to allow return to work; maternal and child health; healthcare provider behavior and TB/HIV.

The largest number of systematic reviews has been conducted on incentive programs, reflecting the increase in results-based financing programs worldwide. We found 75 relevant papers from a review of 181 retrieved from the search. The majority of reviews in this area, 52, have been published between 2011 and 2014. Key health areas that have been focused on include improvements in quality and equity of healthcare provision; tobacco control/smoking cessation; healthcare provider retention and diabetes/chronic illness management.

**Fig. 2:**
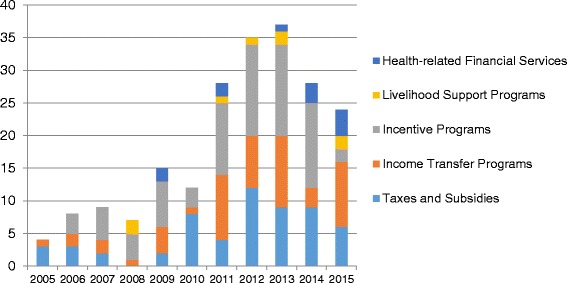
Trends in publications of systematic reviews by economic intervention category. Data projected to end 2015 based on publications from January – July 2015

There was much less evidence from systematic reviews on livelihood support programs and health-related financial services, for which we identified seven and ten systematic reviews respectively. Unlike the other economic intervention categories, the majority of reviews on livelihood support have a fairly narrow disease focus were focused on HIV patients. However, the analysis of changes in the number of systematic reviews over the past ten years (Fig. 2) indicates growing interest in health-related financial services; six of the ten reviews on this topic have been published since 2013.

## Discussion

In order for appropriate and effective economic interventions to be selected by policy makers and program planners, it is important for terminology and intended impact of distinct interventions to be clear, and for existing evidence of impact to be examined. Recognizing the increased interest in economic interventions to improve public health, this paper focuses on proposing a general definition, approach and terminology relating to key economic interventions, and comparing the amount of evidence available from systematic reviews for different categories of economic interventions.

We note that in spite of our proposed overall definition, in some cases, there is no consensus definition of a specific type of intervention, with some definitions focused only on the target population, or some aspect of the intervention design – for instance, microinsurance is used by some to mean any kind of low-cost insurance for the poor, while others use the term microinsurance and community-based insurance interchangeably. This confusion is compounded by the fact that reality is, in fact, complex: the categories above are not always clear-cut. Programs may have numerous (different) interventions at multiple levels. It should also be noted that programs themselves may evolve from one to another for various reasons: for instance conditional cash transfer programs may find that the administrative burden of monitoring conditionality is too high and transform into unconditional programs; or conversely, unconditional cash transfer programs that may not meet their objectives may decide to impose conditions.

The results of our literature review demonstrate that there is a considerable difference between economic interventions in the availability of evidence from systematic reviews. There are far fewer reviews on livelihood support programs and health-related financial services; in order to inform policy makers of the impact of these interventions, this gap in evidence should be addressed by interventional and observational studies as well as by systematic reviews. While the number of reviews on taxes and subsidies, incentive programs and income transfer programs is much higher, there is a notable dearth of reviews focused on health outcomes in low income countries. We also identified an overall lack of information about unintended or perverse outcomes from economic interventions on factors such as equity of service quality and availability, healthcare worker satisfaction, patient satisfaction and healthcare worker time allocation. For example, there is evidence to suggest that one of the world’s largest demand-side incentive programs promoting hospital births through the provision of cash incentives, India’s Janani Suraksha Yojana, not only improved women’s access to services but also increased fertility rates [54]. It is important to consider that impacts of economic interventions – both intended and unintended – often need to be assessed at multiple time points following implementation in order to capture longer-term changes. Finally, we found that data on the implementation, scalability and cost-effectiveness of economic interventions is extremely limited.

## Conclusions

We conclude that while more research and analysis is clearly needed to assess suitability and effectiveness of economic interventions in different contexts, before interventions are tested and further systematic reviews conducted a clear and accurate understanding of the fundamental differences in terminology and approaches is essential among researchers, public health policy makers and program planners. When conducting future assessments of economic interventions, we stress the importance of considering sustainability, scalability, cost-effectiveness and intended consequences.
